# Infectious Agents and Esophageal Cancer: A Comprehensive Review

**DOI:** 10.3390/cancers17071248

**Published:** 2025-04-07

**Authors:** Ahan Bhatt, Hasan Musanna Zaidi, Radhashree Maitra, Sanjay Goel

**Affiliations:** 1 Jacobi Medical Center, Bronx, NY 10461, USA; bhatta4@nychhc.org; 2 Department of Medicine, Albert Einstein College of Medicine, Bronx, NY 10461, USA; 3 Robert Wood Johnson University Hospital, New Brunswick, NJ 08901, USA; hasan.zaidi@rutgers.edu; 4 Department of Medicine, Robert Wood Johnson Medical School, New Brunswick, NJ 08901, USA; 5 Department of Biology, Yeshiva University, Bronx, NY 10461, USA; rmaitra@montefiore.org; 6 Department of Oncology, Montefiore Medical Center, Bronx, NY 10461, USA; 7 Division of Medical Oncology, Rutgers Cancer Institute, New Brunswick, NJ 08901, USA

**Keywords:** esophageal cancer, squamous cell carcinoma, esophageal adenocarcinoma, *Helicobacter pylori*, human papillomavirus, Epstein–Barr virus, herpes simplex virus, cytomegalovirus, oncogenesis, GERD

## Abstract

Esophageal cancer, primarily comprising the squamous cell carcinoma (ESCC) and adenocarcinoma (EAC) subtypes, is the sixth leading cause of cancer deaths globally. In addition to many well-established endogenous and exogenous risk factors, there is emerging evidence for the etiologic role of infectious agents in esophageal cancer, although these associations are incompletely understood. Here, we review the currently available literature on the relationship between infectious agents and esophageal cancer. By far, human papilloma virus (HPV), particularly HPV 16 and 18, have the strongest etiologic association with ESCC. Less robust is the association of high-risk HPV (hr-HPV) with EAC. Although *H. pylori* has been implicated in the development of EAC via increased acid reflux, decreased lower esophageal sphincter tone, and the resultant Barrett’s metaplasia–dysplasia–adenocarcinoma pathway, some hypothesize based on epidemiological trends that *H. pylori* may in fact be a protective factor. In rare cases, EBV can cause esophageal lymphoepithelial carcinoma. Several other agents including HSV, polyomaviruses, and Candida are associated with esophageal cancer to varying degrees. In summary, while several studies, including those conflicting with each other, implicate several infectious agents, the evidence is weak, at best. Clearly, further work is needed to help solidify clear etiologies that will help facilitate prevention and treatment.

## Simple Summary

Esophageal cancer, which includes two main types—esophageal squamous cell carcinoma (ESCC) and esophageal adenocarcinoma (EAC)—is a major global cause of cancer burden, with over 600,000 cases annually contributing to over 500,000 deaths. While many risk factors are well known, researchers are exploring whether infections play a role in its development. For example, the bacteria *H. pylori* might contribute to ESCC by increasing acid reflux and damaging the esophagus, some studies suggest it may actually protect against EAC. High-risk HPV may lead to cancer through genetic disruptions in cell growth. Links between HPV and squamous cell carcinoma vary by region and need more evidence. Rarely, Epstein–Barr virus can cause a specific esophageal cancer type. Other infections, including viruses and fungi, may also have a role, but their impact is not fully understood. This review summarizes current research on how infections may influence esophageal cancer, highlighting the complexity and need for further study.

## 1. Introduction

In 2024, an estimated 22,370 new cases of and 16,130 deaths due to esophageal cancer are projected in the United States [1]. Worldwide, there are annually over 604,100 cases and over 500,000 deaths. Esophageal cancer is more common in males compared to females globally [2]. Histologically, there are two main subtypes of esophageal cancer: esophageal squamous cell carcinoma (ESCC) and esophageal adenocarcinoma (EAC). There is significant variation in the incidence of EC worldwide, partially attributed to dietary, lifestyle, and environmental risk factors in various ethnic subgroups. ESCC is more common in Asian countries compared to the African countries, with EAC more common in Western countries [3]. Over the past few years, the number of cases of EAC is on the rise compared to ESCC, especially in Western countries, with significant decline in the incidence of ESCC [3,4,5].

The etiopathogenesis of esophageal cancer has been well studied and several risk factors have been associated with an increased incidence of esophageal cancer. The esophagus is one of the most common mucosal sites to come in contact with external noxious agents. Additionally, understanding the interaction between the normal healthy tissue and such noxious agents, along with the esophageal microbiome, gives us better knowledge of the causation of esophageal cancer [4,5]. Various infectious agents such as bacteria, viruses, parasites, and fungi are also linked to esophageal cancer [6,7]. They are believed to drive carcinogenesis through chronic inflammation, immune modulation, and direct genetic alteration of the host cells. Human papillomavirus (HPV) and Helicobacterpylori (*H. pylori*) are infectious agents that have been linked with causation of cancers, including esophageal cancer [8]. Significant regional variations also exist, suggesting a role of environmental and genetic factors. However, their role is not yet clearly defined, with conflicting data. Understanding these links can help us further formulate novel preventative as well as therapeutic strategies. Here, we briefly summarize the infectious agents identified in the literature so far, and their role in esophageal cancer.

### Knowledge Gaps

The esophagus is often referred to and considered among the organs as a first line of defense. It is constantly exposed to foreign elements, the most important of which is food to maintain the body. However, as a corollary, it is also exposed to several undesirable entities, including infectious agents, chemicals, and environmental pathogens [9]. Infections, such as viruses, parasites, and bacteria are now well-established causative agents of several cancers. Both proximal and distal to the esophagus, organs develop cancer by an infectious etiology, such as human papillomavirus (HPV), leading to oropharyngeal cancer, and Epstein–Barr virus (EBV), leading to gastric lymphomas [8,10]. Given that the esophagus is at the forefront of exposure to infectious agents, and the limited knowledge of associations between infections and esophageal cancer, we sought to explore the current literature in great depth to serve as a valuable resource to the medical community and the population at large. Drawing attention to infectious etiologies will establish the way for developing strategies for risk mitigation, study of etiopathogenesis, and finally, tailored therapeutics.

## 2. Risk Factors Overview

We see more cases of ESCC compared to EAC in the Asian population. In the US, ESCC is more commonly seen in African Americans and white women, while EAC is more commonly seen in white men [11,12,13].

ESCC has been strongly linked with smoking and tobacco consumption. Moreover, patients with both smoking and alcohol consumption are at greatest risk, with an increase in risk with a higher amount of alcohol intake and years of smoking (*p* < 0.00001) [14,15]. Combined, alcohol and tobacco have a synergistic effect in causing mutations and promoting chronic inflammation [16]. Smokers have a 5-fold risk compared to non-smokers. Additionally, tea, coffee, areca nut (mixed with tobacco), and nitrogenous food products have been reported to be associated with ESCC [15].

EAC is more prevalent among whites and is more common in men compared to women [12]. Additional risk factors associated with EAC include obesity and gastroesophageal reflux disease (GERD), each being independently associated with EAC. High body mass index (BMI) (>25) was directly correlated with increased risk of EAC, while low BMI is associated with ESCC [17]. Lower socioeconomic status is associated with ESCC [12]. GERD, and because of its long-term association with reflux, Barrett’s esophagus (BE), are both associated with EAC [12,18]. BE is considered a precursor lesion of EAC [19,20]. In a large Swedish study, the severity of GERD symptoms was directly associated with the risk of EAC [21]. However, about 50% of EAC is also reported in patients without symptomatic reflux symptoms, which indicate the role of other etiologies [19]. Genetic and other environmental factors are linked with both subtypes of esophageal cancer [5].

The interplay of multiple causal factors has also been studied. A large case control study performed in Korea to investigate smoking, alcohol consumption, and obesity status found alcohol consumption and smoking in age > 55 years to be significantly associated with EC (*p* < 0.001). The study also found BMI > 23 to be protective of EC. In younger patients, age < 55, alcohol consumption is associated with increased risk (*p* = 0.029); however, no association was seen between obesity, smoking and EC. This highlights the potential accumulative synergistic effect of smoking and its causation on EC. The study was limited as it was unable to delineate between ESCC and EAC, where significant differences are deemed to exist [22].

**Figure 1:**
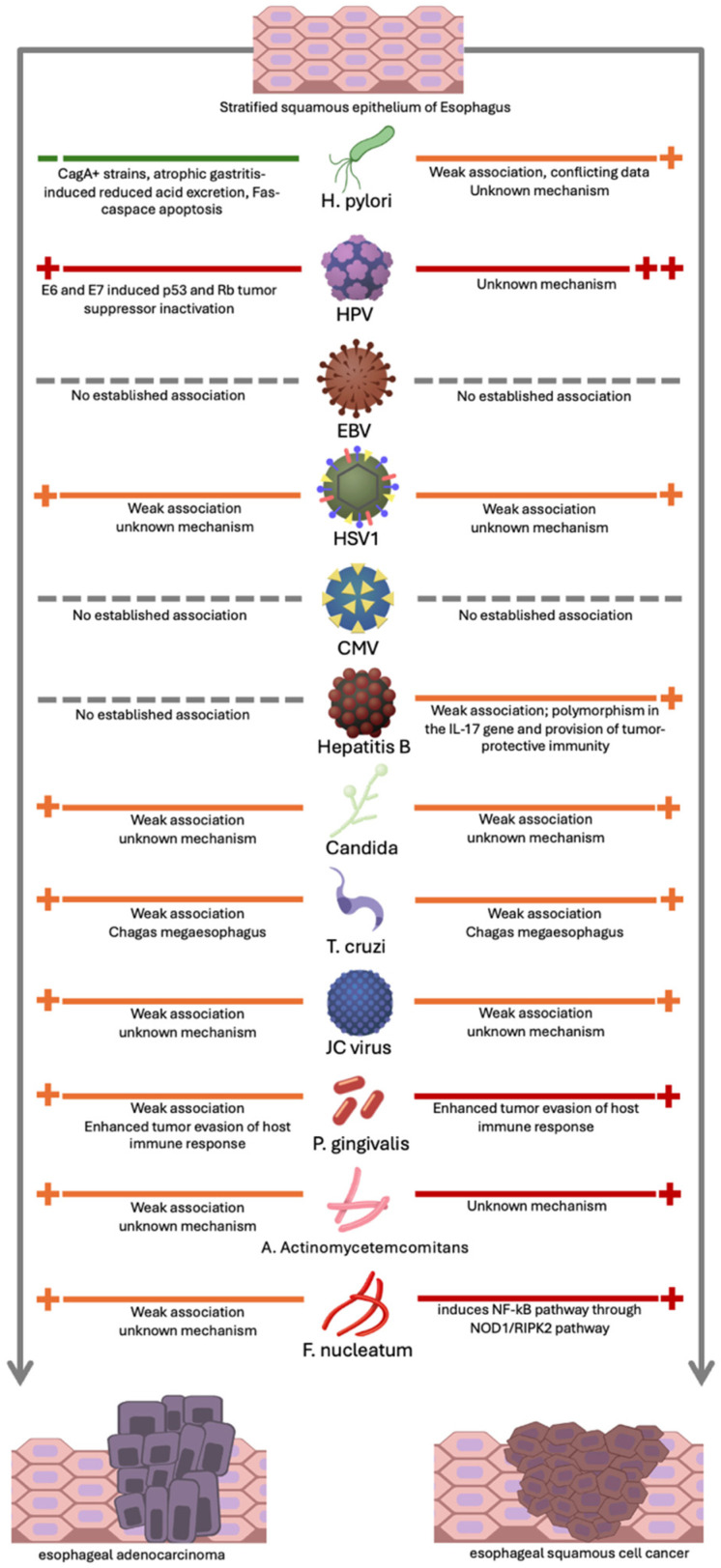
Summary of association of infectious agents with esophageal adenocarcinoma (left) and esophageal squamous cell carcinoma (right). **++** Increased risk (established evidence); **+** Possible risk (moderate evidence); **+** Possible, but limited evidence; **--** No links established; **–** Protective (established evidence).

Several infectious agents are also linked with esophageal cancer [6,7]. The associations are summarized in Figure 1 and are described in detail in their respective subsections in this manuscript.

## 3. *Helicobacter pylori*

### 3.1. Introduction

*H. pylori*, a Gram-negative bacterium, commonly colonizes the stomach, and is linked to peptic ulcers, gastric cancer, and mucosa-associated lymphoid tissue (MALT) lymphoma [23]. Its association with esophageal cancer remains unclear. It asymptomatically infects over half the world’s population, with prevalence varying based on socioeconomic status [24]. Transmission likely occurs via oral–oral, fecal–oral, or environmental routes [25]. Over the last few decades, infections have declined in developed nations due to improved hygiene and sanitation, particularly among younger populations [23,24].

### 3.2. Pathogenesis and Proposed Mechanism for Carcinogenesis

*H. pylori*, primarily implicated in peptic ulcer disease and gastric cancer, is known to survive the acidic environment by penetrating the mucus layer and secreting ammonia and carbon dioxide [26]. The bacterium releases vacuole-forming exotoxin (VacA), inducing cell death, and cytotoxin-associated gene A (CagA), an oncoprotein [27,28]. CagA is delivered into gastric epithelial cells and disrupts vesicular trafficking and autophagy pathways such as the p53 tumor suppressor pathway [29,30].

### 3.3. Association with Esophageal Squamous Cell Cancer

The relationship between *H. pylori* and ESCC remains unclear, although some researchers hypothesize that *H. pylori* might increase ESCC risk through chronic inflammation and increased nitrosamine production [31]. A Swedish case–control study showed serum CagA antibodies and gastric atrophy were associated with an increased risk for ESCC (odds ratio [OR] = 2.1, 95% confidence interval [CI] = 1.1 to 4.0, and OR = 4.3, 95% CI = 1.9 to 9.6, respectively) [31]. A 2002 study showed that *H. pylori* does not induce formation of carcinogenic N-nitrosamines in vitro, which challenges the proposed mechanism linking *H. pylori* to ESCC development [32]. *H. pylori* could, however, be implicated as a secondary factor in the development of ESCC by inducing atrophic gastritis, which reduces gastric acidity and favors the proliferation of other bacteria that do produce carcinogenic nitrosamines [31,33].

Alternatively, some studies have suggested that *H. pylori* plays a protective role. A 2005 study of a Taiwanese population showed that subjects with *H. pylori* seropositivity by ELISA had a lower risk in developing ESCC compared to negative controls when adjusted for factors including age, smoking, and alcohol use (adjusted odds ratio [AOR] = 0.51; 95% CI = 0.27–0.96; *p* = 0.037). *H. pylori* positivity was also significantly lower in patients with lower esophageal ESCC lesions (AOR = 0.34; 95% CI = 0.14–0.80; *p* = 0.013) [34]. Another hospital-based case–control study from Taiwan showed that H pylori infection was significantly inversely associated with ESCC (AOR 0.315–0.472, all *p*-value < 0.05) [35]. However, these results have not been replicated in other geographic regions and may be explained by regional variability in factors such as diet [31].

Two major meta-analyses have investigated the relationship between *H. pylori* infection and ESCC. A 2013 systematic review of 16 studies (1991–2012) found no overall association between *H. pylori* and ESCC (OR = 0.97, 95% CI: 0.76–1.24). However, Eastern populations showed significantly reduced ESCC risk (OR = 0.66, 95%CI: 0.43–0.89), particularly with CagA-positive strains (OR = 0.77, 95% CI: 0.65–0.92). This protective effect was not seen in Western subjects (OR = 1.26, 95% CI: 0.97–1.63) [36]. A larger 2019 meta-analysis (35 studies, 345,886 patients) corroborated these findings, showing no significant general association (OR: 0.84; 95% CI: 0.64–1.09) but demonstrating reduced ESCC risk in Middle Eastern populations (OR: 0.34; 95% CI: 0.22–0.52) [37]. While study heterogeneity was noted due to diverse population characteristics and detection methods, these findings consistently suggest that *H. pylori*’s relationship with ESCC varies by geographic region through mechanisms that remain unclear.

### 3.4. Association with Esophageal Adenocarcinoma

The etiopathogenesis of EAC is primarily linked to GERD and BE, where repeated acid exposure leads to oxidative stress, inflammatory activation, and DNA damage, progressing from metaplasia to dysplasia and eventually to adenocarcinoma [38,39].

Earlier studies suggested the *H. pylori* is actually a risk factor for EAC since it causes increased production of gastrin (a known carcinogenic factor), as well as NF-kB and COX-2 mediated inflammation [40]. However, epidemiological trends suggest that *H. pylori* infection may be protective, since the incidence of EAC in Western countries has been increasing while the prevalence of *H. pylori* infection has fallen [41,42] A 2013 meta-analysis found both *H. pylori* infection in general and CagA-positive strains in particular were associated with lower EAC risk (OR 0.56, 95% CI: 0.45–0.70, respectively) [36]. A 2019 meta-analysis corroborated these findings, showing reduced EAC risk in infected patients (pooled OR 0.55, 95% CI: 0.43–0.70) [37]. This finding was consistent with the results from prior meta-analyses [43,44]. Again, the pooled results were limited by considerable heterogeneity in the source studies, but the heterogeneity was significantly reduced in the subgroup analysis by study area, study type, and H. pylori detection method.

Several mechanisms have been proposed for its protective effect. *H. pylori*-induced gastritis may progress to atrophic gastritis, reducing gastric acid secretion and consequently decreasing BE incidence and halting the metaplasia–dysplasia pathway [45,46,47]. The effect of infection on this process may vary based on the location within the stomach and CagA strain status [43,48,49]. Another possible mechanism is the direct induction of apoptosis in Barrett’s-derived EAC cells by upregulation of the Fas-caspase cascade [50]. Additionally, if *H. pylori* were directly protective from the development of EAC through the Barretts–metaplasia–dysplasia pathway, eradication would likely be associated with worsening GERD/gastritis symptoms as well as EAC itself. And while there has been some variation in findings, studies have generally shown that this is not the case [40,51,52,53,54]. A 2024 Nordic cohort study of 661,987 patients found that *H. pylori* eradication actually decreased EAC risk (SIR 0.73, 95% CI 0.61–0.86) at 11–24 years post-treatment [55].

These findings suggest that *H. pylori* may protect against EAC development in the general population, with significant regional variability and an indefinite mechanism, likely due to factors such as diet and genetics. In summary, H. pylori shows conflicting associations with ESCC and EAC. The potential carcinogenic mechanisms of alteration in gastric acid production and chronic inflammatory changes to the esopahgus, as well as its role in microbiome dysbiosis need to be studied further. We discuss the role of some commensal bacteria in the later sections.

## 4. Viruses

### 4.1. Human Papillomavirus

#### 4.1.1. Introduction

Human papillomavirus (HPV) is a double-stranded DNA virus that infects basal keratinocytes through micro-abrasions in skin and mucus membranes [56]. HPV is divided into low- and high-risk subtypes based on oncogenic potential. Low-risk HPV types can cause a variety of benign cutaneous lesions while high-risk HPV, especially 16 and 18, has shown causal relationship with the vast majority of anogenital SCCs and 26–30% of head–neck cancers [57]. The possible relationship of HPV to esophageal cancer is discussed below.

#### 4.1.2. Epidemiology and Transmission

HPV is widespread, infecting both cutaneous and mucosal epithelium through direct contact. While most infections are asymptomatic, approximately 80% of sexually active Americans will contract HPV during their lifetime [57,58]. Of 2.2 million infection-related cancers globally, HPV accounts for 690,000 cases, primarily cervical carcinoma, with higher prevalence in lower-income countries and immunocompromised individuals [57,58].

#### 4.1.3. Pathogenesis of HPV-Associated Squamous Cell Cancers

The carcinogenic mechanism of HPV has been well studied in anogenital and head and neck cancers. It involves HPV DNA integration into the host genome, leading to increased expression of viral oncoproteins E6 and E7. These proteins inactivate p53 and retinoblastoma tumor suppressors, respectively, through the ubiquitin–proteosome pathway, promoting carcinogenic mutations [59,60,61]. E7’s inactivation of Rb protein results in p16 overexpression, which make it a reliable marker for HPV-positive cancers such as oropharyngeal cancers [62,63].

#### 4.1.4. Association of HPV Status with Outcome to Therapy in Squamous Cell Cancers

HPV positive cancers, like head and neck, cervix, anal canal, have shown significant differences in response to radiation therapy and chemoradiotherapy (ChemoRT) and improvement in outcomes [64,65,66]. Compared to locally advanced HPV-negative oropharyngeal cancer, which had a three-year progression-free survival (PFS) of 38.4%, HPV-positive oropharyngeal cancers had three-year PFS rates of 74.4% [64].

Specifically, in ESCC, Wang et al. showed significant improvement in survival for patients with HPV 16-infected ESCC treated with chemoradiation [67]. The improved treatment outcomes in HPV-positive esophageal cancers can be attributed to viral interference with cellular pathways, essentially promoting error-prone DNA repair pathways, making them more vulnerable to damage from radiation therapy [68]. Additionally, HPV positive cell lines show increased cell cycle arrest in the G2-M phase, which is the most sensitive to radiation therapy [69]. The HPV-infected tumor microenvironment is also hypothesized to be less hypoxic, which increases the efficacy of radiation therapy and is also a possibility [70]. More specifically, in ESCC, presence of p53 mutation in HPV-infected cancer may render it more susceptible to ChemoRT [71]. Future trials evaluating HPV-positive EC 16 and the E7/E6 pathway and response to therapy will help us better evaluate this relationship.

#### 4.1.5. Association with Esophageal Squamous Cell Cancer

HPV’s potential role in ESCC pathogenesis was first proposed in 1982, based on histological similarities between ESCC samples and HPV-associated condylomatous lesions [72,73]. Multiple meta-analyses have since established this association. A 2013 meta-analysis of 21 case–control studies found ESCC cases were three times more likely to have HPV DNA than controls (pooled OR 3.04, 95% CI 2.20–4.20) [74]. Another meta-analysis on high-risk subtypes 16 and 18 specifically showed increased risk of ESCC in HPV-16 infection (OR 3.55, 95% CIs, 2.05–6.14) but not HPV-18 [75]. A 2014 meta-analysis corroborated these findings, showing increased risk with HPV infection, especially HPV-16 (OR 2.69, 95% CI 2.05–3.54 and OR 2.35, 95% CI 1.73–3.19).) [76]. Conversely, Petrick et al. found that HPV prevalence in ESCC cases ranged from 0.176 to 0.322 depending on diagnostic methodology and geography [77]. A 2021 meta-analysis found HPV prevalence in ESCC cases at 18.2% (95% CI 15.2–21.6%) with a pooled OR of 3.81 (95% CI 2.84–5.11) [78]. In general, the association of HPV with ESCC has been consistently demonstrated, but the magnitude of the association has been variable based on many study characteristics. Other meta-analyses failed to show any significant association of HPV with ESCC [79].

Worldwide prevalence of HPV infection in cases of ESCC reportedly ranges from 11.7% to 38.9% with significant geographic variation and HPV detection methodology [79]. High-incidence countries like China and Iran report substantially higher ESCC rates compared to low-incidence countries (approximately 250 vs. 2.5 per 100,000) [80]. Notably, the rate of HPV detection in ESCC cases was also significantly higher in high-incidence countries than low-incidence countries [81]. HPV-ESCC tumor infection rates follow this pattern, with high-incidence areas showing 32.8–63.6% compared to 8.7–16.6% in low-incidence regions like North America [76,77,79]. The meta-analyses on HPV-ESCC association are suggestive of skewed results due to publication bias if the literature reports are primarily sourced from high-incidence countries [79]. For example, in contrast to high-incidence countries like China, low-incidence countries like Korea have not been strongly able to establish a link between HPV and ESCC. In a retrospective analysis of 64 patients with ESCC, only 1 patient was found to be HPV positive. The study also evaluated for other genomic alterations such as Phosphatidylinositol-4,5-Bisphosphate 3-Kinase Catalytic Subunit Alpha (PIK3CA) and potential biomarkers for ESCC such as programmed death ligand-1 (PD-L1) and microsatellite instability/deficient mismatch repair (MSI/dMMR) status in a multivariate analysis, but no statistical correlation could be established due to the low HPV-positive rate in the study. Interestingly, the study did not find any patients with BRAF or KRAS alterations [82].

Detection methodology has been considered as a potential factor in these variations. Contemporary studies primarily use in situ hybridization (ISH) or immunohistochemistry (IHC), showing concordant results (27.7% vs. 24.3%) [77]. Prior detection methods are now considered outdated, such as Southern blotting [81,83]. However, a comprehensive 2013 meta-analysis of 152 studies (10,234 ESCC cases) confirmed that geographic origin, rather than detection methodology, explains the variability in HPV detection rates, with some role of nutrition and environmental exposures [80,81]. The authors pointed out that their results support the hypothesis that HPV may only play a role in ESCC development in high-incidence areas.

Unlike in oropharyngeal and cervical cancers where p16 expression serves as a validated HPV activity marker, its role in HPV-associated ESCC is less definitive [62,63]. A 2014 review found no difference in p16 expression between HPV-positive and HPV-negative ESCC samples [84]. The rate of HPV and p16 double-positivity is approximately 5% in ESCC cases, compared to >90% in HPV-positive oropharyngeal cancers [79]. These findings suggest that if HPV is causally linked to ESCC, the oncogenic mechanism likely differs from other HPV-associated cancers [85,86].

#### 4.1.6. Association with Esophageal Adenocarcinoma

Evidence of papillomatous lesions in the esophagus as well as concurrent increased incidence of EAC along with HPV-associated head and neck cancers have fueled research regarding the potential role of HPV in EAC [87,88]. Meta-analyses showed limited association, with HPV prevalence in EAC samples at 35% (HPV-16 11.4%) and 13%, though these studies were later criticized for methodological bias toward negative results [6,89,90].

A significant breakthrough came in 2013 when Rajendra et al. [91] studied 261 BE/EAC samples, finding that Barrett’s dysplasia and EAC samples were significantly more likely to be HPV-positive compared to controls (IRR 2.94, 95% CI 1.78–4.85 and IRR 2.87, 95% CI 1.69–4.86, respectively). The simultaneous presence of HPV DNA, E6/7 mRNA, and p16 strongly correlated with disease severity [91]. A subsequent study found persistent dysplasia after endoscopic ablation was significantly associated with high-risk HPV (hr-HPV) detection by polymerase chain reaction (PCR) and IHC [92]. These studies identified HPV presence as a predictive factor for both EAC severity and treatment failure, but further studies were necessary to elucidate any direct causal role the virus may play.

Further research in 2016 revealed distinct genomic differences between HPV-positive and HPV-negative EAC samples, particularly in rates of non-silent somatic mutations and TP53 mutations, suggesting a unique oncogenic mechanism [93]. A 2017 study demonstrated that biologically active hr-HPV in Barrett’s dysplasia and EAC exhibited p16 overexpression and reduced/absent pRb and p53, similar to HPV-associated squamous cell cancers. Indeed, the authors found that the combination of high p16 and low pRb expression was identified in a significant proportion of virally positive BD/EAC cases [94].

The most recent meta-analysis confirms HPV’s significant association with increased esophageal cancer risk (both ESCC and EAC) [95]. Therefore, mounting evidence suggests that HPV is associated with EAC in a subset of patients, but more research is needed to establish causality and identify specific risk factors [96]. HPV, despite significant geographical variability, is known to be associated with EC and via mechanisms involving immune evasion and cell cycle dysregulation that can potentiate other causal risk factors. Additionally, no study to-date has studied the role of HPV vaccines. This remains an area of research.

### 4.2. Herpesviruses

#### 4.2.1. Epstein–Barr Virus

##### Introduction

Epstein–Barr virus (EBV, HHV-4) is a member of the human *Herpesviridae* family of linear double-stranded DNA viruses that is nearly ubiquitous in the general adult population, with some studies estimating that >90% of adults will have asymptomatic infection at some point in their life [97]. Transmission of EBV occurs mainly through saliva and respiratory secretions [98]. The virus primarily infects B-cells by binding of the viral envelope to CD21 on the lymphocyte surface but can also infect epithelial cells through various mechanisms [97,99,100].

The most common clinical manifestation of EBV infection is infectious mononucleosis, though it is associated with a broad range of diseases including multiple autoimmune diseases, Alzheimer’s, and Parkinson’s [97]. EBV is also associated with the development of various types of malignancies and has been estimated to contribute to about 1.5% of all human cancers worldwide [101].

EBV-associated malignancies are typically subdivided into lymphoproliferative and epithelial cancers. The lymphoproliferative cancers related to EBV include Burkitt lymphoma, Hodgkin lymphoma, diffuse large B-cell lymphoma (DLBCL), extranodal T/NK cell lymphoma, plasmablastic lymphoma, and primary effusion lymphoma [102]. Among epithelial malignancies, the oncogenic role of EBV has been definitively established in gastric carcinoma and nasopharyngeal carcinoma, with the virus being associated with 8.77% of gastric carcinomas and detected in 95% of nasopharyngeal carcinomas in high-risk regions [103,104,105]. Weaker evidence links EBV to other epithelial malignancies including cervical, renal, thyroid, and bladder cancers as well as lymphoepithelial carcinoma of the salivary glands and lymphoepithelioma-like carcinoma of the lung [97].

##### EBV Oncogenic Mechanism

Like many viruses, EBV has a latent phase after initial infection and a lytic phase that involves destruction of the host cell and release of viral contents. EBV can replicate through two means—proliferation of infected B cells and lytic virion production. The proteins expressed during EBV latency are extensively involved in EBV-mediated oncogenesis. EBV exhibits multiple different types of latency (broadly types I, II, and III), each of which are characterized by a distinct pattern of protein expression. The relevant proteins include latent membrane protein (LMP) 1, LMP 2, Epstein–Barr virus nuclear antigens (EBNA) 1–3, BamHI fragment A rightward transcripts (BARTs), and EBV-encoded small RNA (EBERs), each of which promote oncogenesis through their respective mechanisms which generally involve enhancement of cell survival through disruption of apoptotic pathways, evasion of host immune response, inhibition of cell cycle regulation, and downregulation of tumor suppressors. Specific EBV-related malignancies are associated with particular patterns of latency protein expression. For example, EBV-associated gastric carcinoma and Burkitt lymphoma are associated with type I latency (positive EBNA1, EBER, and BARTs). The details of these particular interactions are discussed extensively in separate reviews [106,107].

##### EBV Association with EAC and ESCC

While EBV has been strongly linked to a subset of gastric carcinoma as mentioned above, its relationship with esophageal cancer is less clear. The evidence on the topic is relatively scare and frequently methodologically limited. Older studies have used PCR, but lately detection of EBERs by in situ hybridization (ISH) is considered the gold standard for establishing EBV presence in a tissue sample [108]. Generally, studies have not shown significant association between EBV and esophageal cancers [90,109,110,111]. Cho et al. in 2001 and Sarbia et al. in 2005 found zero EAC cases positive for EBER by ISH [109,110]. The results were replicated in a 2015 single-center study of gastroesophageal cancers [111]. A subsequent 2017 review and meta-analysis of 30 studies reported that EBV was detectable in only 6% (95% CI 0–27%) of EAC samples, with the entirety of EBV detections occurring by PCR, as the three studies that used solely ISH (referenced earlier) found zero cases of EBV-positive EAC. The studies often had very limited sample size and low geographic generalizability [90]. In contrast, an earlier study by Cho and colleagues found no EBV positivity by EBER ISH in esophageal cancer tumor cells, but did find 5.6% of the samples (8/142) with EBV-positive tumor-infiltrating lymphocytes (TILs) [109]. Collectively, these studies suggest that there is no relationship between EBV and the development of most esophageal cancers (ESCC, EAC), though EBV detection in TILs may be due to a secondary inflammatory reaction and not the etiology of the cancer itself.

##### EBV Association with Lymphoepithelioma-like Cancer

EBV has, however, been more strongly linked to the much rarer primary lymphoepithelioma-like cancer (LELC) of the esophagus. LELC is a poorly differentiated neoplasm characterized by a distinct dense lymphoplasmacytic infiltrate. It is extremely rare and is most commonly reported in China and Southeast Asia [112]. Literature on esophageal LELC exists mainly as case reports, which have been inconsistent. Studies of LELC, particularly of the head and neck and the esophagus, have consistently demonstrated EBV presence by PCR and IHC, but less reliably with ISH [112,113,114,115]. Multiple case reports of LELC found no presence of EBV by ISH, so at this point it is unclear what etiological role EBV may play [116,117,118].

#### 4.2.2. Herpes Simplex Virus

Herpes simplex virus type 1 (HSV-1) has been more linked to esophageal cancer compared to Herpes simplex virus 2 (HSV-2). HSV-1 is commonly transmitted through orolabial mucosa, whereas HSV-2 is more commonly linked with genital lesions. The esophagus is most linked amongst visceral organs, particularly in immunocompromised individuals [119,120]. EC is more commonly linked with HSV-1. The association is not well studied. The potential for an association was originally identified in 1985 with the occurrence of HSV DNA particles in esophageal cancer biopsies [121]. This was further seen by Wu et al. in their study that demonstrated HSV DNA in post-surgical specimens in China [114]. HSV causes non-reflux-mediated esophagitis, which results in a chronic inflammatory state that may be linked with the development of EC, but there is insufficient evidence to establish a direct causal relationship.

#### 4.2.3. Cytomegalovirus

Cytomegalovirus (CMV) infections are usually acquired during perinatal periods or sexual contact. They are known to cause infection in eyes, kidney, and the GI tract, including colitis and esophagitis, especially in the immunocompromised host [122]. CMV esophagitis has been linked to EC [123]. However, no studies have investigated a direct causal link between CMV and EC.

### 4.3. Hepatitis B and Hepatitis C Viruses

Meta-analyses have shown a modest association between chronic hepatitis B and chronic hepatitis C with EC [124,125]. Major evidence, however, is lacking and additional studies are needed to further understand the mechanism of causation. A large epidemiologic study has shown hepatitis B infection to be a positive prognostic biomarker in patients with ESCC, with patients with positive hepatitis B profile experiencing better disease-free and overall survival [126]. It is unclear if this applies to ESCC or EAC or both, since a majority of patients were ESCC. This may be linked to polymorphism in the IL-17 gene and provision of tumor-protective immunity, especially so in ESCC [127].

### 4.4. Polyomaviruses

Polyoma viruses now include several subgroups of viruses. The human polyoma virus or John Cunningham virus is a DNA-based virus that commonly causes progressive multifocal leukoencephalopathy (PML). Very few data exist concerning its association with esophageal cancer. JC virus DNA sequences have been detected in esophageal cancer specimens, among others, and have been labeled as a group 2B carcinogen by the International Agency for Research on Cancer (IARC) [128,129].

## 5. Oral Commensals as Biomarkers

In addition to their role in the pathogenesis of EC, several microorganisms have also been studied as potential biomarkers for screening and prognostic purposes.

### 5.1. Porphyromonas gingivalis

*Porphyromonas gingivalis* was found to play a key role in the origin and development of ESCC. It is a Gram-negative facultative anaerobe and a common commensal in the oral cavity, often a cause of periodontitis and is frequently colonized in the esophageal cavity [130]. *Porphyromonas gingivalis* can also predict disease severity. Elevated serum levels of *Porphyromonas gingivalis* IgG or IgA were associated with worse prognosis of ESCC patients in a research study involving 96 ESCC patients [131]. Additionally, the microbe promotes the spread of EC by upregulating the NF-κB pathway and enhanced expression of epithelial–mesenchymal transition, leading to reduced E-cadherin levels [132,133]. In a mouse model study, it was also found to promote tumorigenesis through HHIP and GLI1 activation—key genes in the Sonic Hedgehog pathway [134]. Additionally, it mediates immune escape by increased Treg production, leading to weakened anti-tumor response through B7-H1 expression [135].

### 5.2. Aggregatibacter actinomycetemcomitans

Along with *P. gingivalis***,** *Aggregatibacter actinomycetemcomitans* is a Gram-negative facultative anaerobe linked with periodontitis, especially in the younger population [136,137]. It has been linked to esophageal cancer (primarily ESCC), pancreatic cancer, and even precursor gastric lesions [138,139,140]. *Aggregatibacter actinomycetemcomitans*, despite being rarely present in healthy individuals, is commonly seen in the oral microbiome in esophageal cancer, and could serve as a screening tool. The bacterium was significantly increased and prevalent from samples in saliva and dental plaques in patients with esophageal cancer, and could potentially be considered a biomarker [138].

### 5.3. Fusobacterium nucleatum

*Fusobacterium nucleatum*, a Gram-negative facultative anaerobe, commonly found in the gut microbiome, invades the ESCC cells and similarly induces the NF-kB pathway, but through the nucleotide-binding oligomerization domain 1 (NOD1)/receptor-interacting protein kinase 2 (RIPK2) pathway, leading to carcinogenesis and progression [141,142]. *Fusobacterium nucleatum* leads to aggressive growth and cell migration along with immune evasion [142]. It is linked to decreased survival in ESCC and could potentially be a marker for prognosis [143,144]. Studies in colorectal cancer also found NF-KB upregulation with activation of mRNA-21 leading to tumor spread [145,146].

The role of oral and esophageal microbiomes and its links to microbial dysbiosis needs to be better understood. The presence of oral commensals also offers the opportunity to study oral samples for better non-invasive screening and prognostication; more research is needed to validate their role to develop biomarkers and/or therapeutic targets.

## 6. Other Infectious Agents

Apart from viruses and bacteria, fungi and parasites have also been sporadically associated with EC. Chronic mucocutaneous candidiasis has also been linked with head and neck cancers and EC in immunocompromised patients [147]. Candidiasis has also been known to be associated with other cancer types, with the majority being asymptomatic infections [148]. Additionally, EC patients are also known to have fungal lesions, ulcerations, and chronic inflammation, potentially linked to fungal ingestion [149]. *Trypanosoma cruzi* has also been linked with gastrointestinal cancer [150,151]. Either by genetic alterations or by increasing GERD, Chagas disease can result in chronic inflammation and potentially increase the risk of EC [152].

## 7. Conclusions

Our review summarizes the role of infectious agents in EC. A simplified overview of our findings is outlined in Table 1 and Figure 1. The strongest association is between HPV (16 and 18) and ESCC, with a less robust association with EAC. HPV produces the oncoproteins E6 and E7 that inactivate tumor suppressor genes (p53 and retinoblastoma protein), leading to the loss of control mechanisms. Further research is needed to study HPV-driven EC and its association with response to treatment, including chemotherapy and radiation therapy, and the role of p16 as a biomarker of HPV-associated EC. Association of H. pylori with EC is less certain; its causative relationship with ESCC varies by geographic region through mechanisms that remain unclear, although it may protect against EAC development. EBV, while not linked to ESCC or EAC, has been more strongly linked to the rare primary lymphoepithelioma-like cancer. Lastly, Candida is linked to development of ESCC. Overall, infections may lead to EC via dysbiosis, inflammation, immune modulation, and production of carcinogens. Further research in identifying and understanding infectious etiologic agents can aid in future strategies for prevention. It will also guide development of novel therapies for the treatment of esophageal cancer.

## Figures and Tables

**Table 1:** Summary of infectious agents associated with the risk in developing esophageal cancer.

Infectious Agent	EAC Risk	ESSC Risk
*H. pylori*	--	+/0
HPV	+	++
EBV	0	0
HSV1	+/0	+/0
CMV	0	0
HBV	0	+/0
HCV	0	0
Candida	+/0	+/0
*Trypanosoma cruzi*	+/0	+/0
Polyoma (JC) virus	+/0	+/0
*P. gingivalis*	+/0	+
*A. actinomycetemcomitans*	+/0	+
*F. nucleatum*	0	+

Index Key: ++: Increased risk (established evidence); +: Possible risk (moderate evidence); +/0: Possible, but limited evidence; 0: No links established; (--): Protective (established evidence).
